# Endoscopic management of leaks and fistulas after bariatric surgery: a systematic review and meta-analysis

**DOI:** 10.1007/s00464-020-07471-1

**Published:** 2020-02-27

**Authors:** Pawel Rogalski, Agnieszka Swidnicka-Siergiejko, Justyna Wasielica-Berger, Damian Zienkiewicz, Barbara Wieckowska, Eugeniusz Wroblewski, Andrzej Baniukiewicz, Magdalena Rogalska-Plonska, Grzegorz Siergiejko, Andrzej Dabrowski, Jaroslaw Daniluk

**Affiliations:** 1 Department of Gastroenterology and Internal Medicine, Medical University of Bialystok, M. Sklodowskiej-Curie 24a, 15-276 Białystok, Poland; 2 Department of Computer Science and Statistics, Poznan University of Medical Sciences, Rokietnicka 7 St. (1st floor), 60-806 Poznan, Poland; 3 Department of Infectious Diseases and Hepatology, Medical University of Bialystok, ul. Żurawia 14, 15-540 Białystok, Poland; 4 Department of Pediatrics, Gastroenterology, Hepatology, Nutrition and Allergology, Medical University of Bialystok, M. Sklodowskiej-Curie 24a, 15-276 Białystok, Poland

**Keywords:** Leak, Fistula, Endoscopic, Bariatric, Sleeve, Gastric bypass

## Abstract

**Background:**

Endoscopic techniques have become the first-line therapy in bariatric surgery-related complications such as leaks and fistulas. We performed a systematic review and meta-analysis on the effectiveness of self-expandable stents, clipping, and tissue sealants in closing of post-bariatric surgery leak/fistula.

**Methods:**

A systematic literature search of the Medline/Scopus databases was performed to identify full-text articles published up to February 2019 on the use of self-expandable stents, clipping, or tissue sealants as primary endoscopic strategies used for leak/fistula closure. Meta-analysis of studies reporting stents was performed with the PRISMA guidelines.

**Results:**

Data concerning the efficacy of self-expanding stents in the treatment of leaks/fistulas after bariatric surgery were extracted from 40 studies (493 patients). The overall proportion of successful leak/fistula closure was 92% (95% CI, 90–95%). The overall proportion of stent migration was 23% (95% CI, 19–28%). Seventeen papers (98 patients) reported the use of clipping: the over-the-scope clips (OTSC) system was used in 85 patients with a successful closure rate of 67.1% and a few complications (migration, stenosis, tear). The successful fistula/leak closure using other than OTSC types was achieved in 69.2% of patients. In 10 case series (63 patients), fibrin glue alone was used with a 92.8–100% success rate of fistula closure that usually required repeated sessions at scheduled intervals. The complications of fibrin glue applications were reported in only one study and included pain and fever in 12.5% of patients.

**Conclusions:**

Endoscopic techniques are effective for management of post-bariatric leaks and fistulas in properly selected patients.

**Electronic supplementary material:**

The online version of this article (10.1007/s00464-020-07471-1) contains supplementary material, which is available to authorized users.

Bariatric–metabolic surgery remains the most effective method of obesity treatment providing long term weight loss and improvement of obesity-related diseases. According to the International Federation for the Surgery of Obesity and Metabolic Diseases, the number of bariatric procedures performed worldwide in 2013 exceeded 460,000. The most commonly performed bariatric procedures are gastric bypass (GB), sleeve gastrectomy (SG), and laparoscopic adjustable gastric banding (LAGB) [1, 2].

Although bariatric procedures are effective, they have various degrees of success and complication profiles that are unique to the procedure type. Overall, bariatric surgery has a low incidence of serious complications of approximately 4% and mortality rate of 0.1% [3, 4]. The Agency for Healthcare Research and Quality (AHRQ) and recent clinical studies report significant improvements in metabolic and bariatric surgery safety, which is mainly associated with the increased use of laparoscopy and advances in surgical techniques [3, 4] Among all complications, fistulas and leaks are major adverse events which increase post-operative morbidity and mortality, especially in the acute phase [5]. The incidence of leaks after SG has been reported to be approximately 1.06% [6]. Post-SG leak can lead to the development of gastric fistula over time. Fistulas after SG occur in 0.2% to 2.5% of cases and are most commonly located at the proximal third of the gastroplasty [6, 7]. Leaks are also the major complications of GB, occurring in 0.7% to 5% of patients. They are usually located at the gastrojejunal anastomosis, but have also been noted at the distal esophagus, gastric pouch, remnant stomach, blind jejunal limb, and jejunojejunal anastomosis [8, 9].

The treatment of fistula/leak may involve surgical, endoscopic, and/or radiological procedures [3]. Over the last years, the management evolved with the development and improvement of several endoscopic techniques including self-expanding metal (SEMS) and plastic stents, clipping techniques [including the use of through the scope clips (TTSC) and over-the-scope clips (OTSC)], tissue sealants, suturing systems (OverStitch System®), and internal drainage techniques [3, 10–12]. The use of endoscopic therapies has gained popularity over time and tends to be more standardized among expert teams. The available literature contains many case reports, case series and only a few retrospective observational cohort studies assessing the use of different endoscopic techniques to treat post-bariatric leaks and fistulas. The results of these studies are inconclusive as they report the use of individual techniques as monotherapy or in combination with surgery or other endoscopic technique, mainly based on a given center’s experience. There are no prospective, randomized studies on this topic. Meta-analyses and systemic reviews on this topic are also limited [13–15].

With this in mind, we performed a meta-analysis and systematic review of self-expanding stents, clipping techniques, or tissue sealants used as primary strategies in the treatment of leak/fistula after SG, GB, and LAGB with the aim to assess technical characteristics, successful closure rate, and technique-related complications.

## Materials and methods

This systematic review and meta-analysis was performed in accordance with the guidelines formulated in the Cochrane Handbook for Systematic Reviews of Interventions [16]. The authors followed the Preferred Reporting Items for Systematic reviews and Meta-Analyses (PRISMA) guidelines for systematic reviews and meta-analyses.

### Search strategy

A systematic literature search was conducted by two researchers (R. P. and S-S. A) to identify and appraise studies of endoscopic management of anastomotic leaks and fistulas after bariatric surgery. MEDLINE (PubMED) and SCOPUS databases were searched from inception to February 2019. The formulas used to search the MEDLINE (PubMED) and SCOPUS databases are showed in Supplementary Materials. The reference lists of review articles were hand-searched for additional relevant studies. Inclusion and exclusion criteria were determined by two researchers (R. P. and S-S. A). All authors independently determined studies eligible for meta-analysis and systematic review. Institutional review board approval and written consent for this paper was not required.

### Definitions

Based on the initial review of the literature, we found that both the definition of leak and the time intervals (acute, early, late, chronic) varied. There was also no universal definition of fistula. While some authors defined it as late or chronic leak, others used the term leak and fistula interchangeably. Therefore, in the inclusion criteria, we used the general definition of leak and fistula as an endoscopic or radiologically confirmed dehiscence of anastomosis or leakage of gastrointestinal content from a surgical join between two hollow viscera or through a suture line around an organ or the presence of a luminal content collection next to the anastomosis [17].

### Eligibility criteria

Included studies employed trials involving endoscopy in the management of anastomotic leaks and/or fistulas after bariatric surgery. Only full-text articles, focused on self-expanding metal stents or tissue sealants or clipping techniques, published in English were considered. Randomized controlled trials, non-controlled clinical trials, observational cohort studies, and case series (≥ 3 cases) were considered eligible for the meta-analysis. The inclusion criteria were as follows: (1) research on patients with fistulas and leaks after bariatric procedures, including gastric sleeve, gastric bypass, or laparoscopic adjustable gastric banding, (2) studies in which the stent or clipping technique or tissue sealant application was the preferred method of endoscopic leak/fistula closure, (3) studies in which stents were used after previous unsuccessful attempts at endoscopic or surgical treatment. The following studies were excluded: (1) studies evaluating only combined endoscopic methods; (2) studies without clear data and/or description of therapy are used; and (3) studies focusing on endoscopic methods other than the stent or clipping technique or tissue sealant application. In addition, a meta-analysis of studies on self-expanding stents included only the studies specifying the frequency of stent migration.

### Extracted data and subgroup analysis

The following data were extracted: (1) study characteristics (author name, publication year, type of study); (2) data on participants (sample size, age, gender); and (3) data on interventions, success, and adverse events of endoscopic therapy.

Data on stents, clipping techniques, and tissue sealants were analyzed and presented separately. A meta-analysis of studies reporting the use of stent in the treatment of leak/fistula was performed to assess the frequency of successful leak/fistula closure (defined as the percentage of patients with successful leak/fistula closure, confirmed by endoscopy or contrast X-ray after), and stent migration. In addition, systemic reviews of studies reporting the use of clipping techniques and tissue sealants were performed to determine the efficacy and complications of such therapies.

### Statistical methods

A random-effects model described by DerSimonian and Laird was used to aggregate the study data [18]. For zero-score events, the continuity correction was performed by adding a correction factor of 0.5. Proportions of overall successful leak/fistula closure, stent migration as well as successful leak/fistula closure in gastric sleeve and gastric bypass group were given with 95% confidence intervals that are based on exact binomial Clopper–Pearson method [19]. Statistical heterogeneity between the studies was evaluated with the Q Cochrane’s statistics and the *I*^2^ coefficients, which showed contribution of heterogeneity relative to the whole for each study. The publication bias was examined by visual inspection of funnel plots and formally with Begg’s test with continuity correction [20]. Furthermore, sensitivity analysis was performed for parameters showing significant heterogeneity. The analysis was performed using the STATA software, version 14.2 (forest plot and Begg’s test) and PQStat software, version 1.8.0 (visual interpretation of funnel plot and sensitivity analysis). The significance level of 0.05 was assumed.

The efficacy of clipping techniques or tissue sealants was presented as a percentage of leak/fistula closure and the frequency of technique-related complications.

## Results

**Fig. 1:**
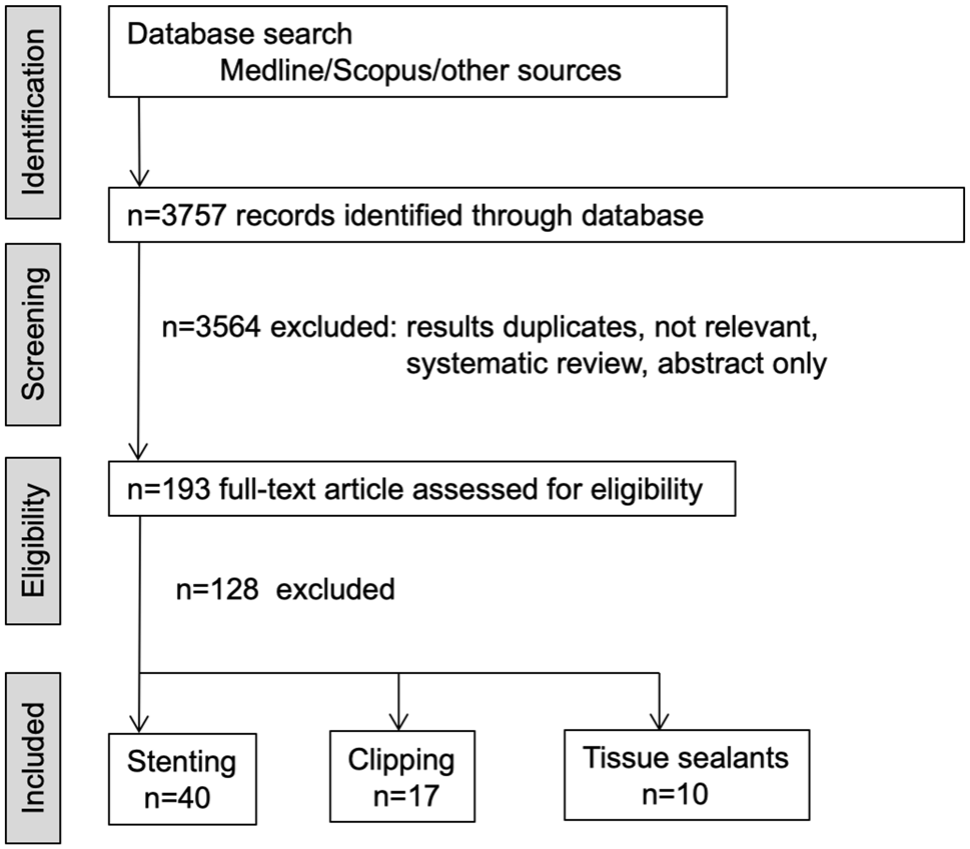
Flowchart for search strategy and selection of eligible studies for systemic review and meta-analysis

The initial database search identified 3757 reference articles, in which 65 relevant articles were selected and reviewed (Fig. 1 and Supplementary material: Figure S9 show the search results).

### Self-expanding metal and plastic stents

Data focused on the efficacy of self-expanding stents in the treatment of leaks and fistulas after bariatric surgery were extracted from 40 studies (493 patients) that met the inclusion criteria. We did not find randomized controlled trials and non-controlled clinical trials. Therefore, only cohort observational studies and case series were included in the meta-analysis. All selected studies were published between 2006 and 2019 (Table 1) [21–61].

**Table 1:** Studies assessing self-expanding stents in the treatment of post-bariatric leak/fistula eligible for meta-analysis

Study	Population (n)*	Bariatric surgery SG (n)/GB (n)	Stent type
Krishnan et al. (2019)	31	16/15	EndoMaxx silicone-coated, plastic, covered
Al Lehibi et al. (2018)	3	2/1	Niti-S MEGA, SX-ELLA esophageal
Emre et al. (2018)	4	4/0	Hanarostent Esophagus Bariatric Surgery
Klimczak et al. (2018)	13	13/0	Niti-S MEGA
Tsai et al. (2018)	5	5/0	Taewoong Niti-S
Boerlage et al. (2018)	36	13/23	Niti-S Beta
Almadi et al. (2017)	64	64/0	WallFlex fully covered esophageal, Niti-S covered esophageal, polyflex esophageal
Garofalo et al. (2017)	7	7/0	Wallstent, Megastent
Tringali et al. (2017)	8	8/0	Niti-S Beta
Montuori et al. (2017)	5	5/0	FCSEMS Beta (Taewoong Medical)
El-Sayes et al. (2017)	16	16/0	Niti-S FCSEMS Esophageal
van Wezenbeek et al. (2016)	12	7/5	Hanarostent ECBB
van den Berg et al. (2016)	8	2/6	Hanaro CCI FCSEMS
Aydın et al. (2016)	4	4/0	Hanaro
Rebibo et al. (2016)	9	9/0	Hanarostent
Quezada et al. (2015)	29	19/10	FCSEMS
Périssé et al. (2015)	29	23/6	Boston Scientific SEMS
Matlok et al. (2015)	3	3/0	WallFlex Easophageal Stent
Vix et al. (2015)	7	7/0	Hanarostent ECBB
Fishman et al. (2015)	26	26/0	Hanarostent and Megastent
Moon et al. (2015)	6	6/0	–
Alazmi et al. (2014)	17	17/0	Ultraflex Boston Scientific
Galloro et al. (2014)	4	4/0	Megastent, Taewoong
Aras et al. (2014)	3	3/0	UBPS, SEMS
Leenders et al. (2013)	11	6/5	Hanarostent, Choo stent, Endoflex
Freedman et al. (2013)	35	0/35	Danis Stent
Simon et al. (2013)	9	9/0	Hanarostent stent Taewoong stent
Marr et al. (2012)	4	4/0	Wallflex
Corona et al. (2012)	6	6/0	Wallflex fully covered esophageal stent,
Yimcharoen et al. (2011)	9	6/3	Alimax-E or Evolution or Ultraflex or Polyflex
Inbar et al. (2011)	3	3/0	SX-ELLA esophageal stent (ELLA-CS)
de Aretxabala et al. (2011)	4	4/0	FCSEMS
Tan et al. (2010)	8	8/0	FCSEMS
Blackmon et al. (2010)	10	4/6	Alimax-E
Nguyen et al. (2010)	3	3/0	Alimax-E, Wallflex
Casella et al. (2009)	3	3/0	Ultraflex and NITI-S Esophageal Stents
Edwards et al. (2008)	6	0/6	Polyflex, Boston Scientific
Fukumoto et al. (2007)	4	1/3	Polyflex, Boston Scientific
Eisendrath et al. (2007)	12	4/8	Ultraflex, Silky Esophageal Stent
Salinas et al. (2006)	17	0/17	Ultraflex

*SG* sleeve gastrectomy, *GB* gastric bypass, *UBPS* uncovered biodegradable polydioxanone stent, *SEMS* self-expandable metal stent, *FCSEMS* fully covered self-expandable metal stent

^*^Only data on GB and SG patients have been analyzed

The median body mass index (BMI) of patients varied between 32 (30–42) and 56.6 (44–65) kg/m^2^. Sixteen studies provided information on the interval between surgery and leak diagnosis; this period ranged from 4.6 (3–7) to 142.3 (7–252) days. The time between leak diagnosis and stent placement was reported by 6 studies and ranged from 5 (3–10) to 82 (5–367) days. The time between surgery and stent placement was reported by 6 studies and ranged from 14 (7–21) to 95 (13–395). The median interval between implantation and removal of the stent was reported in 22 studies and varied between 15 (14–16) and 121.7 (18–341) days. Leaks were most often located within the gastroesophageal junction (GEJ) near the proximal end of the staple line or in the distal portion of the esophagus, or in the upper third of the gastric stump. The mean estimated defect size was reported only in one study and was 1.18 cm [38].

#### Overall proportion of successful leak closure

**Fig. 2:**
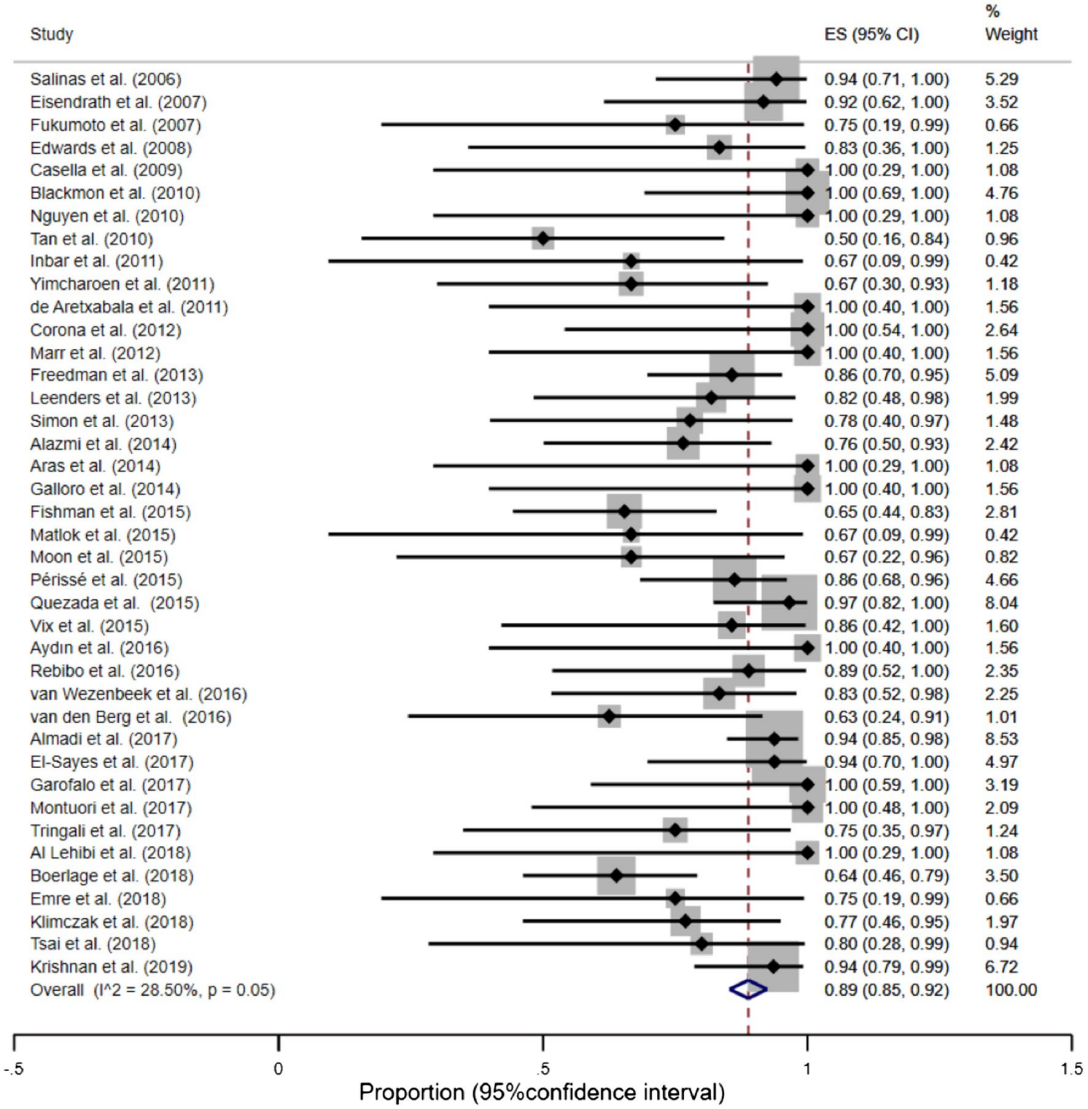
Forest plot for successful leak closure. After exclusion of outliers: overall proportion of successful leak closure = 92% (95% CI, 90–95%), test of heterogeneity *I*^2^ = 0.00% (*p* = 0.77)

The overall proportion of successful leak/fistula closure was 89% (95% CI, 85–92%) (Fig. 2). However, the funnel plot, sensitivity analysis and the Begg’s test suggested an existing bias and asymmetry between the studies (Supplementary material: Figure S1). Therefore, several studies were excluded [48, 52, 62]. After excluding above studies, the remaining studies were homogenous (*I*^2^ = 0.00%, *p* = 0.77) and the overall proportion of successful leak or fistula closure did not change significantly—92% (95% CI, (90–95%) (Supplementary material: Figure S2).

#### Successful leak closure in gastric sleeve group

**Fig. 3:**
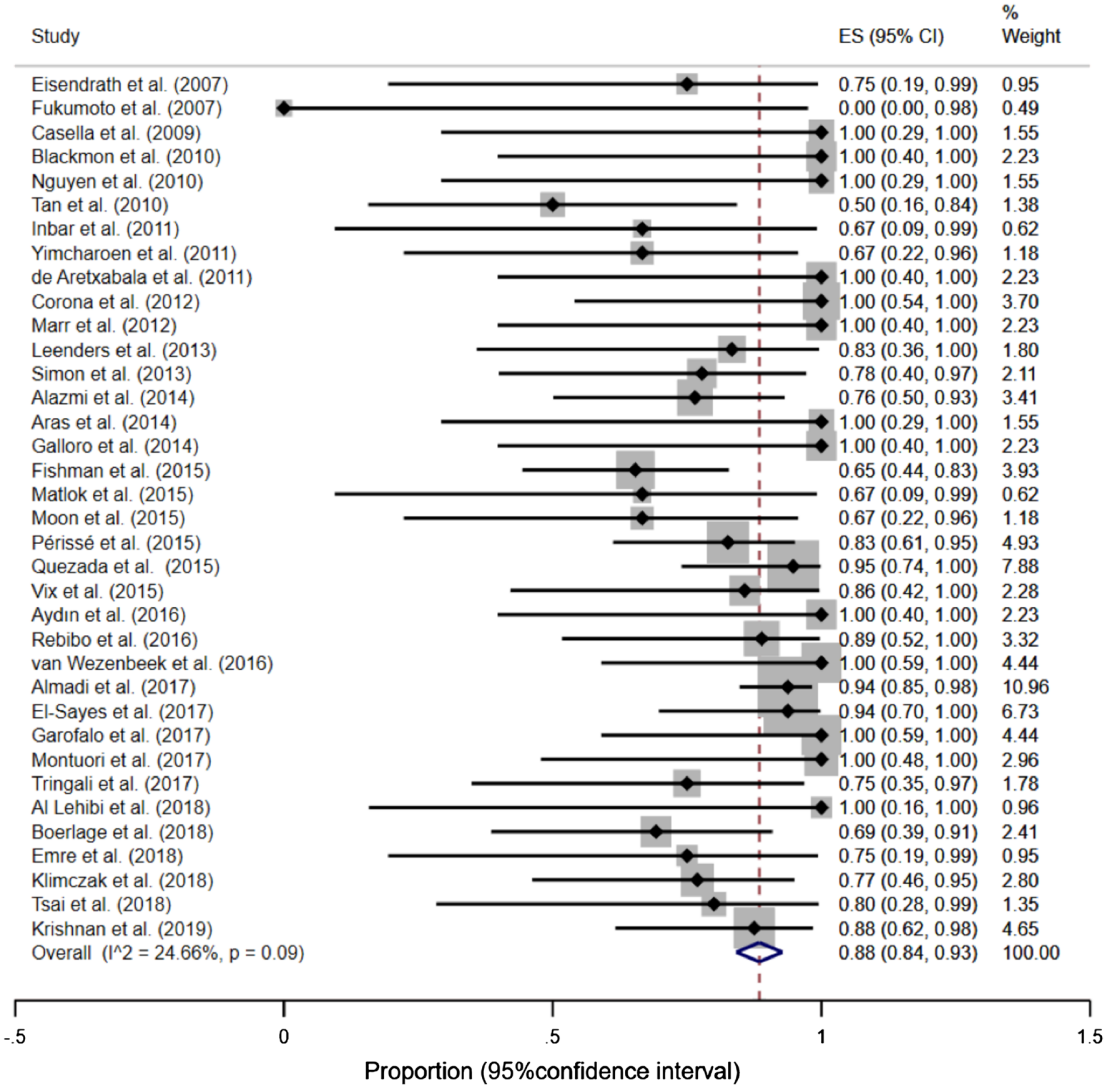
Forest plot for successful leak closure in gastric sleeve group. After exclusion of outliers: overall proportion of successful leak closure = 92%, (95% CI, 88–95%), homogeneity coefficient was *I*^2^ = 0.00% (*p* = 0.81)

Thirty-seven studies (Table 1) reported the effectiveness of SEMS after gastric sleeve (344 patients). The proportion of successful leak closure in gastric sleeve group was 92% (95% CI, 88–95%), *I*^2^ = 0.00% (*p* = 0.81)—Fig. 3, Supplementary material: Figures S3 and S4.

#### Successful leak closure in gastric bypass group

**Fig. 4:**
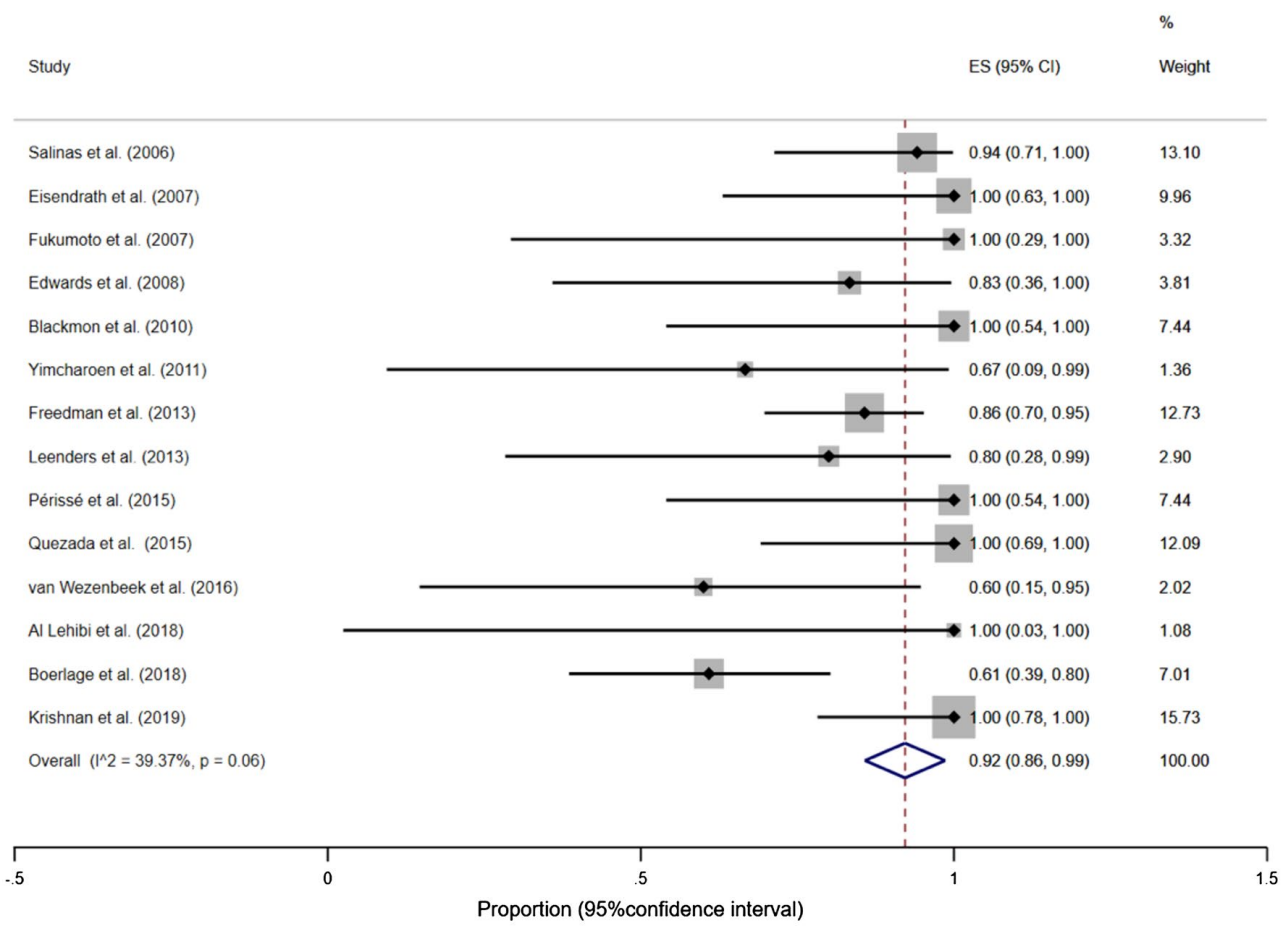
Forest plot for successful leak closure in gastric bypass group. After exclusion of outliers: overall proportion of successful leak closure = 96%, (95% CI, 91–100%), homogeneity coefficient was *I*^2^ = 0.00% (*p* = 0.58)

Fifteen studies (Table 1) reported the effectiveness of SEMS after gastric bypass (149 patients). The proportion of successful leak closure in gastric bypass group was 96%, (95% CI, 91–100%), *I*^2^ = 0.00% (*p* = 0.58)—Fig. 4, Supplementary material: Figures S5 and S6.

#### Stent migration

**Fig. 5:**
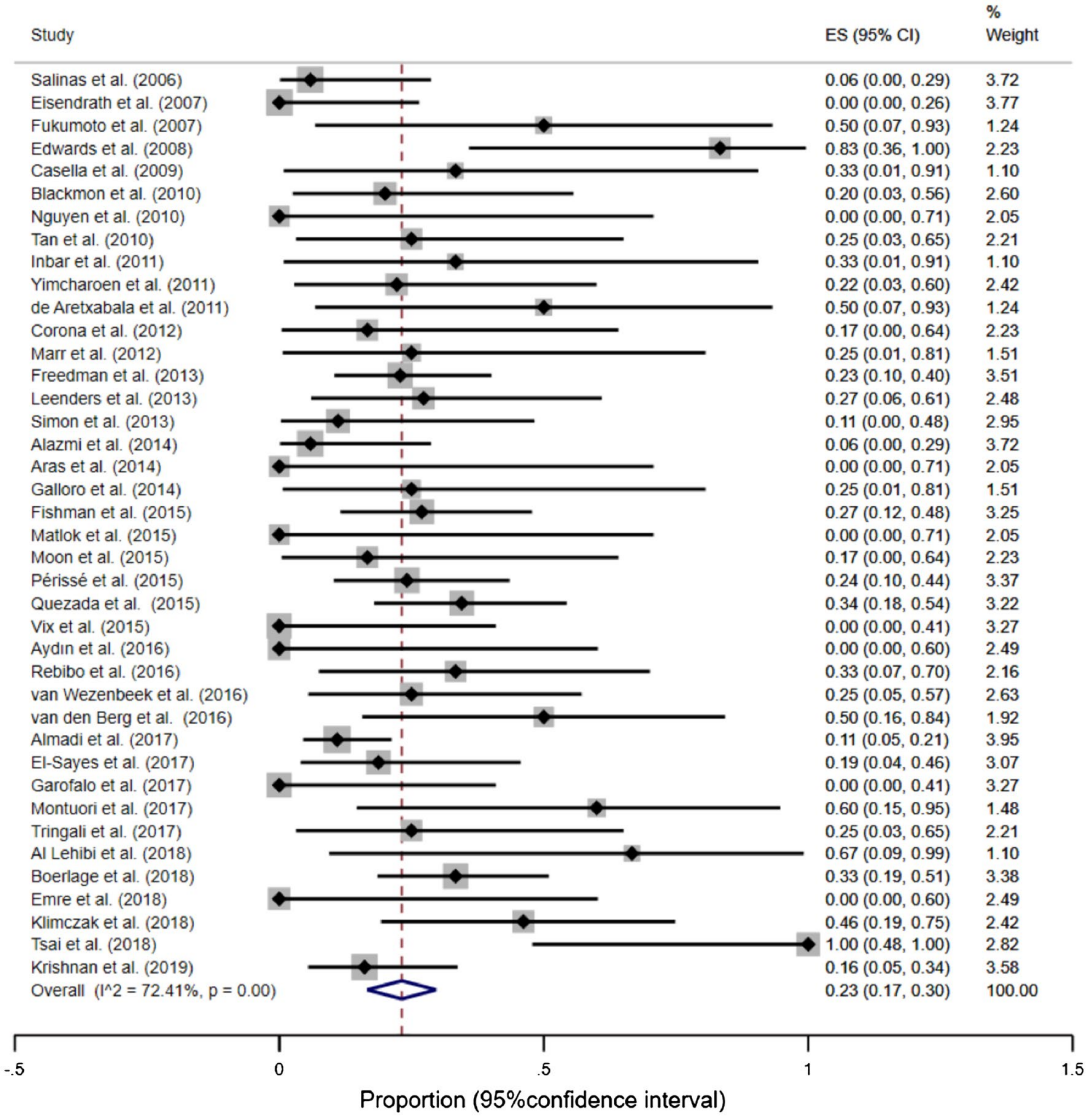
Forest plot for stent migration. After exclusion of outliers: overall proportion of stent migration = 23% (95% CI, 19–28%), test of heterogeneity I^2^ = 3.36% (*p* = 0.41)

The overall proportion of stent migration was 23% (95% CI, 17–30%) (Fig. 5). However, a significant heterogeneity between the studies was observed (*I*^2^ = 72.41%, *p* < 0.01). Also, the funnel plot, the sensitivity analysis, and the Begg’s test suggested an existing bias and asymmetry (Supplementary material: Figures S7 and S8). Therefore, several studies, which contributed to heterogeneity the most and were located outside the funnel, were excluded [21, 23, 24, 26, 34, 38, 40, 63]. After excluding the above studies, the remaining studies appeared to be homogeneous (*I*^2^ = 3.36%, *p* = 0.41) and the overall proportion wasn’t changed, but the precision increased and the confidence interval become narrower—proportion of stent migration = 23% (95% CI, 19–28%). The contribution of individual studies to the total proportion of stent migration was comparable.

### Clipping techniques

We identified a total of 46 papers by our initial search protocol and performed a manual review for relevant articles describing the use of clips in the treatment of leak/fistula after bariatric therapies (Supplementary material, Figure S9) [40, 53, 64–91]. Among them, we found 17 articles reporting clipping as the preferred endoscopic therapy for leak/fistula closure. There were seven case reports including a total of 9 patients and ten retrospective studies including a total of 89 patients. Additional therapies used are noted in Table 2. Patients age varied between 21 and 67 years. The leak and/or fistula were the complications of SG in seven studies, GB in nine studies, and LAGB in two studies.

**Table 2:** Clipping in the management of post-bariatric leak/fistula—characteristics of studies, included patients, and leak/fistula occurrence

Study	Study type (n)	Study information/additional treatmet (n)	Clips alone (n)	Bariatric surgery	Age (range)	M/F	Time from surgery to leak/fistula	Symptoms
Tabibian/2017	CR (1)	Combined radiological-endoscopic approach	1	GB	65	0/1	NA	Leak, intraabdominal abscess
Callabero/2016	CR (1)		1	SG	29	1/0	8 days	Abdominal pain
Donatelli/2014	CR (1)	Revision surgery as the first treatment, persistence of fistula 2 months later	1	GB	43	0/1	4 years	Weight regain
Aly/2013	CR (2)	Laparoscopic drainage, persistence of leak after 6 weeks (1)	2	SG	58 44	0/2	8 days, 11 months	Pain (2), fever (2), peritonitis (1)
Ritter/2013	CR (2)	Gastrojejunal anastomosis leak – percutaneous drainage Gastrogastric fistula due to ulcer—surgical revision, persistence of leak 2 months later	2	GB	43 44	0/2	11 days 6 years	Pain (2), fever (1), collection (1), malaise (1)
Iacopini/2010	CR (1)	Band removal (erosion) and external drainage (26 months after placement)	1	LAGB	45	0/1	2 weeks after band removal	Pain, abdominal abscess
Merrifield/2006	CR (3)	Persistence of fistulas (diagnosed 10 days after GB) after percutaneous drainage	1	GB	NA	NA	10 days	Pain, sepsis, abdominal abscesses
Lee/2018	RS (21)	Different etiology (3 cases after bariatric surgery)	3	NA	47, 49, 32	1/2	3–4 days	NA
Benosman/2018	RS (26)	Comparison of endoscopic drainage (pigtail catheter with and without other endoscopic techniques, n = 7): + OTSC: 3, OTSC + SEMS: 2, + SEMS: 2 vs endoscopic closing techniques alone: (n = 19): OTSC: 7, OTSC + SEMS: 5, SEMS: 7 Prior therapy: external drainage (10, 45.5%)	7	SG	45.6 (± 8.9)	7/19	NA	NA Abdominal collection in 84.6% of patients
Kim/2017	RS	Complications of LAGB in 26 patients (7.4%) including intragastric migration, gastric leaks and fistulas. Endoscopic treatment in 6 patients including gastric leaks (n = 2) and fistulas (n = 1) 1 patient with leak treated with OTSC required percutaneous drainage of the fluid collection	1	LAGB	37	0/1	15 months	Abdominal pain, band site infection
Niland/2017	RS (14)	Prior therapies: surgical revision (4), APC (11) Concomitant therapy (1)	14	GB	46–67	3/11	0–38 years (mean: 9.4 years)	Abdominal pan (7), nausea/vomiting (6), weight gain (2), heartburn (2)
Keren/2015	RS (26)	OTSC (19/26) Other (7/26): stent (6), plastic biliary prosthesis (1), argon cautery (1), biologic glue (1)	19	SG	21–60	9/10	3–21 days	Septic shock (1), tachycardia (3), fever (6), pain (5), collection (3), none/routine contrast study (4)
Law/2015	RS (47)	Different etiology, bariatric surgery (10) (8 GB)	8	GB	NA	NA	NA	NA
Moon/2015	RS (15)	Conservative treatment: 8 (then stent in 6, surgery in 3) Endoscopic treatment: 5 (fibrin glue in 3, hemoclips in 2) Clips used also as second therapy in failures	2	SG	22–55	2/13	Early leak (1–6 weeks)	Nausea, pain, fever
Mercky/2014	RS (30)	Different etiology, bariatric surgery: 19/30 (18 SG, 1 GB) Treatment: OTSC (15/19); OTSC + SEMS: (3/19), OTSC + SEMS + clips + glue (1/19)	15	SG GB	25–59	6/13	Early fistulas: 5 (7–20 days) Chronic fistulas: 14 (50–1100 days)	NA
Surace/2011	RS (19)	Bariatric surgery: 12 (11 SG, 1 GB)	12	SG GB	NA	NA	NA	NA
Bhardway/2010	RS (8)	All fistulas treated with clips and APC	8	GB	22–66	0/8	< 1 year: 2 5–15 years: 6	Weight regain (5), nausea (3), vomiting (2), pain (1)

*n* number, *CR* case report, *RS* retrospective, *OTCS* over-the-scope clip, *SG* sleeve gastrectomy, *GB* gastric banding, *SEMS* self-expandable metal stent, *APC* argon plasma coagulation, *NA* data not available

The time interval between surgery and diagnosis of leak/fistula was reported in 13 studies and ranged from 1 day to 38 years. Among them, 10 studies reported leak/fistula occurrence within a month after bariatric procedure. The clinical manifestations of leak/fistula were shown in 13 studies (53 patients) and included abdominal pain, fever/peritonitis, nausea/vomiting, and abdominal collections. Weight regain as the main clinical symptom was reported by 8 patients (4.2%) (Table 2). In 1 study, 4 of 19 patients did not report any symptoms and fistula was diagnosed in control imaging examinations [75].

The location of leak/fistula was described in all except one study. The most common locations of leak/fistula were proximal staple line, gastrogastric, and gastroesophageal junction (Table 3). In addition, there were three gastrocutaneous fistulas and one esophagobronchial fistula reported by one study [77]. The size of the leak/fistula was assessed by six studies and varied between 3 and 20 mm. Thirteen studies (85 patients) used the OTSC system for leak/fistula closure and only one system was required in most of the studies. The size of OTSC was specified in five studies (16 OTSC in 13 patients) and included: 12/6t (8 OTSC), 12/6gc (6 OTSC), 14/6t (1 OTSC), and 11/6t (1 OTSC). Four studies (13 patients) reported the use of other endoclips including TriClip, Quick Clip, and Resolution Clips (1–6 clips per patient) [53, 68, 70, 79]. Prior therapies used before clipping included external drainage (seven studies), revision surgery (three studies), argon plasma coagulation (one study) (Tables 2 and 3). In addition, we presented the data regarding OTSC therapy from the study describing the use of endoscopic closing techniques alone versus endoscopic drainage (with or without other endoscopic techniques) [72]. We excluded from our analysis studies or study results reporting the concomitant use of stent or stent placement before clipping (Supplementary material, Figure S9) [40, 66, 70, 72, 75, 77, 78, 81–91].

**Table 3:** Clipping in the management of post-bariatric leak/fistula—characteristics of leak/fistula and endoscopic procedures

Study	Localization (n)	Size (mm)	Clip type (size)	Success and failure	Complications of clipping	Follow-up (time and imaging)
Tabibian/2017	Gastric pouch	NA	OTSC (12/6t)	Yes	–	2 days (swallow test) and 4 months Stable body mass, no symptoms
Callabero/2016	Proximal staple line	5	OTSC (12/6t-10 mm)	Yes	–	6 months (endoscopy), 12 months (examination/endoscopy) After 1 year asymptomatic, weight loss remained above 75%
Donatelli/2014	Gastrogastric	NA	1 OTSC (14/6t)	Yes	–	30 days (UGI series) Weight loss
Aly/2013	GEJ (1) GEJ (1)	NA	OTSC (12/6t)	Yes	–	1 case—2 weeks (endoscopy) 8 months; 2 case—6 months (blue dye test)
Ritter/2013	Gastrojejunal anast. (1) Gastrogastric (1)	NA	2 Endoclips	Yes	–	14 days (UGI series)
Iacopini/2010	GEJ (2 fistulas)	NA	OTSC and OTSC + stent	Yes		24 h (barium swallow), 8 weeks (barium swallow)
Merrifield/2006	Gastrogastric and Gastroperitoneal	NA	1 TriClip + 4 Qucik Clip	Failure after 20 days: 2TriClips + APC At 6 weeks: closure of peritoneal fistula, persistence of small gastrogastric (PPI)		20 days and 6 weeks (endoscopy, UGI series) 1 year—clinical assessment
Lee/2018	Anastomotic esophagogastrostomy site	5–10	OTSC	Technical success: 3/3 Final success: 2/3 Failure: 1/3	None	750–840 days
Benosman/2018	Proximal staple line	8.3 (± 4.6)	OTSC	Success of OTCS alone: 7/7	None related to OTSC Complications related only to stent placement	Mean follow-up: 122 days (± 72) Every 3–4 weeks endoscopy Recurrence diagnosis with CT scan and endoscopy
Kim/2017	Posteroinferior site		OTSC	Success of OTCS: 0/1 Further treatment: SEMS and surgery	None	UGI contrast series
Niland/2017	Gastrogastric	3–15	OTSC (Multiple attempts with OTSC in 3 patients)	Technical success: 12/14 Lost of follow-up: 4/14 Primary success (normal UGI series/subsequent endoscopy): 5/10 Long term success (status on recent imaging): 3/4 (mean 6.6 months)	None	Mean: 6.6 months (UGI series and/or endoscopy)
Keren/2015	GEJ (18) Antral (1)	NA	1 OTSC (21) 2 OTSC (5)	Success (since clip till full oral diet initiated, range: 14–60 days): OTCS: 17/19 Failure OTCS: 2/19	–	Endoscopy
Law/2015	Gastrogastric	NA	OTSC (12/6GC: 6 12/6 T: 4, 11/6 T: 1)	Immediate clinical success: 6 (75%) Need for repeated intervention: 5 (63%) Delayed clinical success (after 8 weeks): 4 (50%)	NA	Median follow-up: 178 days (IQR: 63–326) Suspicion of recurrent fistula: endoscopy, radiologic imaging
Moon/2015	GEJ	NA	Hemoclips	Yes	NA	NA
Mercky/2014	Esophagogastric (14) Gastrocutaneous (3) Gastrogastric (1) Esophagobronchial (1) Enterocutaneous (1)	3–15	OTSC	Primary success (technical and clinical after first procedure): OTSC: 9/15; OTSC + SEMS: 2/4 Secondary success (with additional treatment after initial OTSC failure): OTSC: 4/15; OTSC + SEMS:1/4 Failure: OTCS: 2/15; OTSC + SEMS: 1/4	Anchor migration (1), fistula edges lorn by anchor (1), mediogastric stenosis caused by OTSC (1)	NA
Surace/2011	SG: gastric GB: gastrogastric	NA	OTSC	Primary success after SG: 6 (54.5%) (in 1 case with 2 OTSC) Secondary success after SG: 4 (36.5%) Failure: 1 case after SG, 1 case after GB	1 complication related to delivery system (the anchor blocked within the clip)	Mean follow-up: 8 months
Bhardway/2010		< 20 mm	3–6 Resolution clips	Early failure (after 2 weeks): 2 Delayed failures (after several weeks): 2 Further treatment in failures: surgical repair	None	At 2 weeks: UGI series Maximum follow-up: 8–46 months

*N* number, *GEJ* gastroesophageal junction, *SG* sleeve gastrectomy, *GB* gastric bypass, *OTSC* over-the-scope clip, *SEMS* self-expandable metal stents, *APC* argon plasma coagulation, *PPI* proton pump inhibitor therapy, *UGI* upper gastrointestinal imaging, *NA* data not available

Different definitions of therapy success were applied in the reviewed studies. Overall, successful closure of a leak/fistula with the OTSC system was achieved in 57 of 85 patients (67.1%). However, five studies reported the need for additional treatments after primary OTSC therapy to achieve secondary success such as: multiple attempts with OTSC [74, 76], other endoscopic therapies (SEMS, standard clips, glue, suturing) [73, 76, 77, 80], and surgery [73, 76, 80]. Only two studies reported complications related to the OTSC system including anchor migration (1 patient), mediogastric stenosis (1 patient), and one complication related to delivery system (anchor blocked within the clip) [77, 80]. Among four studies describing the use of other clips for leak/fistula closure, successful treatment was reported in 9 of 13 patients (69.2%) (Table 3). In one study, 4 of 8 patients (50%) with therapy failure were referred for surgical repair [79].

The follow-up imaging included radiology and/or endoscopy in all studies and was performed in different intervals varying from 2 days to 12 months. Post-closure follow-up ranged from 14 days to 46 months (Table 3). Three case reports provided information about follow-up body weight and reported weight loss in two patients and stable body mass in one patient [64–66].

### Tissue sealants

We found 10 case series comprising 63 patients treated by the application of tissue sealant—fibrin glue—as a single endoscopic method for leak/fistula closure after bariatric surgery (Tables 4 and 5). The time between bariatric operation and sealant application varied from 1 to 144 days (median for 6 reports: 12.5 days) [92–97].

**Table 4:** Tissue sealants in the management of post-bariatric leak/fistula—characteristics of studies, included patients, and fistulas

Study	Study type, n	Study information/additional treatment (n)	Sealant, n	Bariatric surgery	Age (years)	M/F	Localization of fistula (n)	Size (mm)	Symptoms
Assalia A/2018	RS (24)	Percutaneous application under endoscopic control, first choice method of endoscopic treatment in this center	24	SG	18–53, mean 42	10/14	GEJ (24)	NA	Fever 83.3% pain 66.6 dyspnea 25% cloudy drainage from drain 16.6%
Brolin R/2013	RS (13)	8 leaks after laparoscopic RYGB, and 5 leaks after revision, 2 treated conservatively with TPN and drainage, 5 reoperated, 6 treated by fibrine glue from endoscopic access, 1 treated by reoperation and then fibrin glue	7	RYGB	28–60 mean 46.6	0/13	GE (5), pouch (2)	NA	NA
Papavramidis/2001	CR (2)	Last application in patient treated with 6 sessions was done by percutaneous fistuloscopy done with choledochoscope	2	VBG, vertical gastroplasty with artificial pseudopylorus	41,59	1/1	Near pylorus (2)	NA	Sepsis–1 pt Discharge from drainage tube–1 pt
Papavramidis S/2004	CR (3)	TPN, somatostatin in all	3	Vertical gastroplasty or biliopancreatic diversion with vertical/lateral gastrectomy	25–16	3/0	Duodenojejunal junction (2) or non-defined (1)	NA	Sepsis– 3 pts
Papavramidis S/2008	CR (6)	TPN, somatostatin and PPI in all. In 3 patients conservative treatment was successful	3	SG or biliopancreatic inversion with duodenal switch	28,37,43	2/1	Duodenojejunal anastomosis (6)	NA	Fever- 1 pt 2 pts- NA
Kowalski Ch/2017	CR (8)	Out of 8 patients, 5 was treated endoscopically, other 3 were operated because of unstable vital signs. All patients treated endoscopically had the tip of external drain found in the gastric stump	5	Laparoscopic RYGB	51,42,33, 40,43	0/8	Gastrojejunal anastomosis (8)	NA	NA
Garcia-Caballero M/2005	CR (1)	TPN, octreotide Tip of external drain found in the gastric lumen	1	One anastomosis gastric bypass	36	0/1	Gastrojejunal anastomosis (1)	NA	Routine contrast study
Ece I/2015	CR (2)	TPN, drain tip in the gastric pouch	2	RYGB	31,50	0/2	NA	Drain size	Saliva in drain dischrge
Kotzampassi K/2015	RS (63)	63 pts with fistulas after many operation types, TPN, endscopic or mixed endoscopic and percutaneous access	14	NA	NA	NA	NA	NA	NA
Kim SY/2017	CR (6)	Out of 6 patients 2 treated with sealants: 1st with fibrin glue and 2nd with histoacryl	2	GB SG	37,24	0/2	NA	NA	Epigastric pain

*n* number, *CR* case report, *RS* retrospective, *SG* sleeve gastrectomy, *GB* gastric banding, *VBG* vertical banded gastroplasty, *RYGB* Roux-en-Y gastric bypass, *NA* data not available

**Table 5:** Tissue sealants in the management of post-bariatric leak/fistula—characteristics of endoscopic procedures

Study	Sealant, no of patients	Amount of sealant per session	No of sessions	Intervals between sessions	Success and failure	Complications of sealing	Time from surgery to endoscopic treatment	Time from first sealing to fistula closure	Interval between treatment and discharge	Follow-up (time and imaging)
Assalia A/2018	(24) Fibrin glue, Evicell	5 ml	1–9 pts, 2–8 pts, 3–3 pts, 5–2 pts, 6–1 pts	2 weeks	23/1 (95.8%)	Pain (2), fever (1)	The fistula was acute in 10 patients, subacute in 9 and chronic in 5	1–90, Median 22 days	NA	42.3 months (range 20–46)
Brolin R/2013	(7) Fibrin glue, Tisseel	Large amounts	1–3 pts, 2–1 pts, 3–3 pts	3 days	7/7 (100%)	None	1 day-3 months, median 3 days	NA	Mean 27 days in the operated group and 18 days in fibrine glue group	NA
Papavramidis/ 2001	(2) Fibrin glue, Beriplast	2 ml	1–1 pt, 6–1 pt	2 days	2/0 (100%)	None	12 days, 20 days	2, 34 days	NA	1 pt-24 months gastroscopy, 2 pt- NA
Papavramidis/2004	(3) Fibrin glue, Beriplast	2–4 ml	3–2 pts, 9–1 pt	2 days	3/0 (100%)	None	18, 7, 7 days	18, 48, 14 days	NA	NA
Papavramidis/ 2009	(3) Fibrin glue Beriplast	Large amounts	3–1 pt, 6–2 pts	2 days	3/0 (100%)	None	11, 5, 144 days	15, 16, 26 days	17, 15, 25 days	NA
Kowalski Ch/2007	(5) Fibrin glue,Tisseel	10 ml	1–4 pts, 2–1 pt	NA	5/0 (100%)	None	5,7,9,15,33 days	Mean 4 days	3, 4, 4, 7, 10 days	12 months
Garcia-Caballero M/2005	(1) Tissucol	4 ml	1	–	1/0 (100%)	None	14 days	4 days	28 days	24 months
Ece I 2015	(2) Tisseel	4 ml	1–2 pts	–	2/0 (100%)	None	14 days	5 days	7,9 days	NA
Kotzampassi K/2015	Fibrine glue (Tissucol, Beriplast), Histoacryl	2 ml	Fibrin glue: 1–14, median 4; Histoacryl: 1–9, median 4	2–3 days	13/14 (92.8%)	None	8 days	NA	8–32, mean 14 days	3 months
Kim SY/2017	(1) Fibrin glue, Greenplast; (1) histoacryl	2 ml	1	–	2/0 (100%)	None	1st pt-NA 2nd pt-2 weeks	1,6 days	NA	NA

*NA* data not available

In 8 reports (25 patients), the sealant was delivered via endoscopic access with a 100% success rate in fistula closure. Some authors passed a cytology brush into the fistula orifice to clean away debris and loose granulation tissue before application of fibrin glue [98]. In 2 studies (38 patients) the sealant was delivered via combined percutaneous and endoscopic access with 95.8–92.8% rate of fistula closure [96, 98] (Table 4).

The number of sessions per patient needed for successful fistula closure was reported in 9 studies comprising of 49 patients [92–95, 97–101]. Success was achieved in 48 patients: after 1 session in 22 (45.82%) patients, after 2 sessions in 10 (20.83%) patients, after 3 sessions in 9 (18.75%) patients, after 5 sessions in 2 (4.17%) patients, after 6 sessions in 4 (8.33%) patients, and after 9 sessions in 1 (2.08%) patient (Table 5). One study reported only a median number of 4 sessions needed for closure [96]. In the majority of reports the sessions of sealing were repeated every 2 to 3 days. Only in a report from Assalia et al. [98] were sessions scheduled at 2 week intervals.

The exact volume of fibrin glue used per session was reported in 6 studies and ranged from 2 to 10 ml (median 4 ml). In 6 reports, endoscopic treatment was combined with a total parenteral nutrition and in addition with somatostatin or octreotide in 3 reports [93–97, 100].

The complications of fibrin glue applications were reported in only one study and included pain and fever in 3 of 24 patients (12.5%) [98].

## Discussion

In our systematic review and meta-analysis, we focused on the most commonly used endoscopic therapies such as self-expanding stents, clipping, and tissue sealants used as the preferred endoscopic method for leak and/or fistula closure. In general, we found a high efficiency of self-expanding stents, clipping techniques, and fibrin glue in closing post-bariatric leak/fistula.

The effectiveness of self-expanding stents was the subject of two previously published meta-analyses [13, 14]. Eight years have passed since the publication of the first of them. Therefore, it summarizes the preliminary results of the use of stents non-strictly designed for the treatment of leaks and fistulas after bariatric surgery. In addition, the significance of this meta-analysis is limited by the relatively small sample size. A second meta-analysis included publications from 2006 to 2016 presenting more recent results. However, out of the 28 studies included in this meta-analysis, only 4 studies used stents designed specifically for treating leaks after bariatric surgery. Such stents were used in 11 studies included to our meta-analysis. Seven of these studies were published in 2017–2019. The use of stents dedicated to bariatric patients is becoming a standard therapy approach, therefore, our meta-analysis better reflects the current results of treatment of post-bariatric leaks with self-expanding stents. In fact, the results of endoscopic stent treatment obtained in our meta-analysis were significantly better compared to the previous meta-analysis 92% versus 72.8% and 96% versus 76.1% in GS and GB group, respectively. We can speculate that this was due to more frequent use of stents designed to treat post-bariatric leaks. Unfortunately, most publications did not provide results of the effectiveness of closing leaks and fistulas in relation to the stent used. Therefore, we were unable to perform a separate analysis.

Clipping techniques including the OTSC system were also effective for leak/fistula closure. The overall successful closure was achieved in 67% of patients. In the previously published systematic review, 86.3% of patients treated with the OTSC system had an overall successful leak/fistula closure [15]. However, it included studies with concomitant or previous additional endoscopic procedures such as stent placement. Therefore, the results of this systematic review demonstrate the effectiveness of endoscopic combined techniques rather than the clips themselves. In our systematic review, an overall successful leak/fistula closure was significantly lower compared to previous systematic review. However, we did not analyze studies reporting concomitant stenting or other endoscopic methods. Moreover, Shoar et al. analyzed the effectiveness and safety of the OTSC system only, while in our study we looked at other clips systems.

The most commonly used sealant for fistula closure is fibrin glue—a tissue-compatible adhesive working in a double manner. It mechanically occludes the stomach wall defect and plays a predominant role in wound healing, inducing cellular response to tissue damage, forming matrix-building strands, which promote neovascularization and fibroblast proliferation [102]. Some bariatric surgeons routinely use fibrin sealant to facilitate healing of stapled closures and anastomoses as a prevention of leaks [103]. In our study, fibrin glue was highly effective, but in most cases repeated sessions were necessary to achieve final closure. The main reasons of tissue sealing failure was due to the huge orifice of the fistula [95] or non-compliance of the patient who did not appear regularly on scheduled sessions of fibrin glue application [98]. Although the cost or the fibrin glue is considerable, the cost of one session of tissue sealing is more than five times lower than that of stent insertion and more than 6 times lower than OTSC [95]. Among other types of tissue sealants there are cyanoacrylate glue and SurgiSIS [99, 104]. Cyanoacrylate, a synthetic glue working as a mechanical sealant, has the advantage of having high adhesive and high antibacterial properties, and thus is suitable for application in infectious sites. It is eliminated by hydrolysis after a significant time period (1–6 months), and only a small quantity of the glue is needed [95]. The cost of one portion of cyanoacrylate is approximately six times lower than one portion of fibrin glue [95]. Despite these advantages, the poor mechanical properties of the film, brittle nature, possible proinflammatory effect as well as the risk of damage of the endoscope because of its rapid polymerization make cyanoacrylate a second-choice method. There are fewer studies on those sealants than on fibrin glue, usually combining multiple techniques. Thus, we were unable to extract the specific effectiveness of tissue sealant instead of the effectiveness of other methods or combined endoscopic therapy from these studies [82]. Kotzampassi et al. demonstrated high efficacy of 96.8% of cyanoacrylate in a heterogeneous series of 8 patients with fistulas after various types of surgery [95]. The volume of cyanoacrylate delivered was 0.5 or 1 ml for one session. Total volume applied was 0.5–4 ml (median 1.5) in a median of 2 sessions (range 1–4 sessions). However, the study does not provide detailed information about the type of surgery or fistula location. On the other hand, Vilallonga et al. reported high efficiency of transcutaneous application of cyanoacrylate in combination with stent implantation in the treatment of gastrointestinal fistulas [104]. SurgiSIS (Wilson-Cook, Winston-Salem, NC) is an acellular matrix biomaterial formulated from the porcine small intestine submucosa that stimulates proliferation and formation of fibroblasts in the region of wounds and incorporates into the scar without initiating a foreign body inflammatory reaction. Strips of soaked SurgiSIS material are captured within a specially designed polypectomy snare and loaded into the endoscope outside of the patient. This quite inconvenient delivery method is balanced by high efficacy. The rate of 5 to 20 mm wide fistulas closure after 3 sessions was achieved in 20 of 25 patients [99].

The use of endoscopic therapies demands precise visualization of the internal fistula orifice, which can be a great challenge. In many patients, its presence is confirmed late, after numerous radiologic and endoscopic examinations. Proper selection of patients seems to be critical for favorable outcomes. Patients qualified to endoscopic therapy were hemodynamically stable and in many cases the leak was controlled by percutaneous drainage. Septic patients with uncontrolled gastrointestinal leaks or peritonitis should be treated surgically. The success of endoscopic therapies in the management of leak/fistula also depends on the defect’s size. In general, self-expanding stents allow closing the largest leaks and fistulas. The studies reported use of clipping including the OTSC system for fistulas not larger than 20 mm. In addition, thin and clean fistula canals facilitated quick closure with fibrin glue application. However, most studies lacked information about fistula orifice size. Due to the heterogeneity of the endpoints of the included studies, we were also not able to perform additional analyses on: the optimal time to start endoscopic therapy, and the length of endoscopic treatment. Some of the studies provided information on the duration of stent dwelling time. Although the time range was very wide, the period of maintaining stents in the gastrointestinal tract recommended by most authors was 6–8 weeks.

To ensure the clarity of results, we did not include studies assessing complex endoscopic techniques. However, reviewing the publications from recent years, we found some excellent research on the endoscopic treatment of fistulas and leaks after bariatric surgery [84, 105–107]. These studies show an important trend in the treatment of post-bariatric leaks. Currently, complex endoscopic surgical treatment or combined treatment with simultaneous or sequential use of several endoscopic methods seems optimal in management of the complications of bariatric surgery. Therefore, future research should focus on assessing the effectiveness of complex therapies rather than individual endoscopic methods. Rebibo et al. compared the results of endoscopic management of large gastric leaks or gastric stenoses associated with gastric leaks using covered stents with endoscopic combined treatment using covered stents and double-pigtail stents [51]. The median time to gastric leak closure was shorter, the number of endoscopic procedures, the stent migration rate and the failure rate was significantly lower in the double-pigtail plus covered stent group compared to the covered stent group only. Shebab et al. performed a retrospective analysis of 81 patients with leaks after SG or GB treated with Mega stent (an ultra-large covered stent) alone or in combination with OTSC, which was applied in 46% of patients [84]. The OTSC was placed simultaneously with the stent or after stent removal. The final leak closure was achieved in 82% of patients with a mean of three endoscopic procedures per patient. The authors concluded that the approach combining stents with OTCS is highly effective, but Mega stents should be used with caution as most of observed complications were associated with stent placement. The authors mentioned that clip placement in the treatment of post-bariatric leaks is less effective as the leaks are surrounded by fibrotic and inflamed tissue and clips can act as a foreign body limiting healing. Therefore, they preferred the use of stent as the first method, and clips when the leak persist after stent therapy [84].

Cost-effectiveness is another important issue related to the treatment of post-bariatric complications. Unfortunately, no cost analysis was carried out in any of the studies included in our systematic review and meta-analysis. Theoretically, the use of endoscopic methods can contribute to reducing the costs associated with reoperation and the patient's stay in the ICU. However, the cost-effectiveness of individual endoscopic methods also varies. Cosse et al. have recently shown that double-pigtail stents for the treatment of gastric leak is more cost-effective than covered stents and should be proposed as the standard regimen whenever possible [105].

We did not find endoscopic procedure-related mortality. In our meta-analysis, the frequency of stent migration, which is the most common self-expanding stent-related complication was 23%. In contrast, two recently published meta-analyses reported stent migration rates to be 16.94% and 30.5% [13, 14]. The use of endoscopic suturing or clip application to fix esophageal stents and prevent migration have been reported only in a few studies. Therefore, we were not able to perform a comparative analysis between studies that used or did not use these techniques. The analysis of the relationship between stent type and frequency of stent migration was not possible due to the large variety of stent types used. Nevertheless, based on the individual results of some studies, it seems that the use of partially covered stents reduces the risk of migration. These stents increase tissue hyperplasia which can complicate their removal. Other severe complications of stent implantation were rare. One reported gastrointestinal perforation [38]. Two studies showed minor bleeding as a complication of stenting [32, 34]. Some patients reported dysphagia and vomiting as a result of esophageal stenosis due to tissue hyperplasia, obstruction of the stent with food, or stent collapse [21, 27, 34, 37, 40]. Mild retrosternal discomfort, nausea, excessive salivation which resolved spontaneously after a few days were the most commonly reported symptoms associated with stents [21, 37, 41]. The first published studies reported difficulties in stents removal [21, 24]. Nevertheless, stent removal is currently easily performed due to the improvement of stent design and stent removal techniques.

Clipping or fibrin glue – related complications were also rare, although reported only by a few studies without adequate follow-up information. Only one study reported pain and fever in 12.5% of patients after fibrin glue application [98]. Two studies described a few complications associated with clipping such as anchor migration, tear, mediogastric stenosis, and one complication related to the delivery system (anchor blocked within the clip) [77, 80].

Our study has several limitations. First, due to the high heterogeneity of the included studies and missing data, we could not analyze some of the assumed endpoints. The funnel plot, sensitivity analysis and the Begg’s test suggested an existing bias and asymmetry between the studies assessing stents. Therefore, several studies had to be excluded from the meta-analysis. Moreover, our analysis was limited by the inclusion of retrospective studies, case series, and case reports due to lack of prospective, randomized controlled trials. There is also a risk of bias associated with the publication of only those studies in which the effectiveness of stents, clips and tissue sealants has been confirmed. Several retrospective studies reported the efficacy of leak/fistula closure using different techniques, probably dependent on the experience and capabilities of medical centers. In addition, reports describing the clipping techniques or fibrin glue in the treatment of leak/fistula used different definitions of therapy success and follow-up time. And finally, we included studies on post-operative leaks as well as chronic fistulas, so the timespan from surgery to diagnosis of the defect was wide, as well as the timespan from the diagnosis of leak to stent implantation. Interestingly, all reviewed methods were shown to be effective in both acute leaks as well as chronic fistulas.

In summary, to the best of our knowledge, our meta-analysis and systematic review is currently the largest analysis of the efficacy and safety of endoscopic treatment of post-bariatric leaks and fistulas.

In conclusion, there is the most evidence of the effectiveness of self-expanding stents in the treatment of post-bariatric leaks and fistulas. However, despite the use of new stent designs, the frequency of stent migration remains high. Post-bariatric fistulas and leaks with an orifice size of up to 20 mm can also be successfully treated with clips, preferably OTSC. In turn, application of fibrin glue allows closing narrow fistulas. However, it may require multiple sessions to achieve leak closure. There is an urgent need for RCTs to assess the efficacy and safety of both individual as well as combined endoscopic methods in the treatment of post-bariatric leaks and fistulas.

## Electronic supplementary material

Below is the link to the electronic supplementary material.

Figure S1. Funnel plot, sensitivity analysis, and the Begg’s test result for successful leak closure (PNG 346 kb)—

Figure S2. Forest plot - successful leak closure after exclusion of outliers (PNG 468 kb)

Figure S3. Funnel plot, sensitivity analysis, and the Begg’s test result for successful leak closure in gastric sleeve group (PNG 248 kb)

Figure S4. Forest plot successful leak closure in gastric sleeve after exclusion of outliers (PNG 396 kb)

Figure S5. Funnel plot, sensitivity analysis, and the Begg’s test result for successful leak closure in gastric bypass group (PNG 204 kb)

Figure S6. Forest plot successful leak closure in gastric bypass group after exclusion of outliers (PNG 201 kb)

Figure S7. Funnel plot, sensitivity analysis and the Begg’s test result for stent migration (PNG 280 kb)

Figure S8. Forest plot for stent migration after exclusion of outliers (PNG 383 kb)

Figure S9. Search flowchart of studies on the use of clipping for leak/fistula closure (PNG 309 kb)

Electronic supplementary material 2 (DOCX 63 kb)
