# Supplementary figures and images for: Endoscopic management of leaks and fistulas after bariatric surgery: a systematic review and meta-analysis
Source: Surg Endosc. 2020 Feb 27;35(3):1067–87. doi: 10.1007/s00464-020-07471-1 (PMC7886733; doi:10.1007/s00464-020-07471-1)

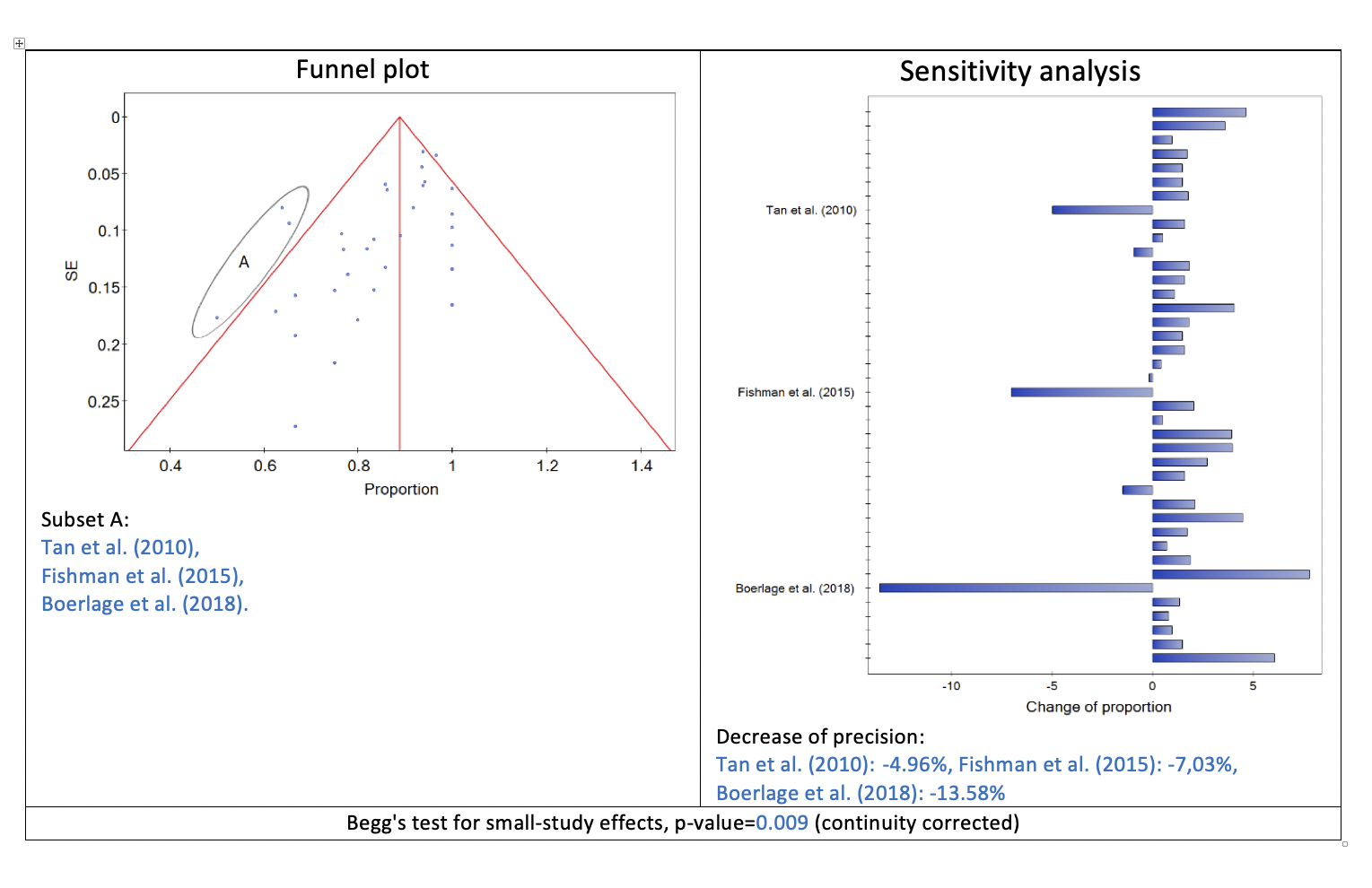

Supplement: Supplementary file 1 — Figure S1. Funnel plot, sensitivity analysis, and the Begg’s test result for successful leak closure (PNG 346 kb)— [file 464_2020_7471_MOESM1_ESM.png]

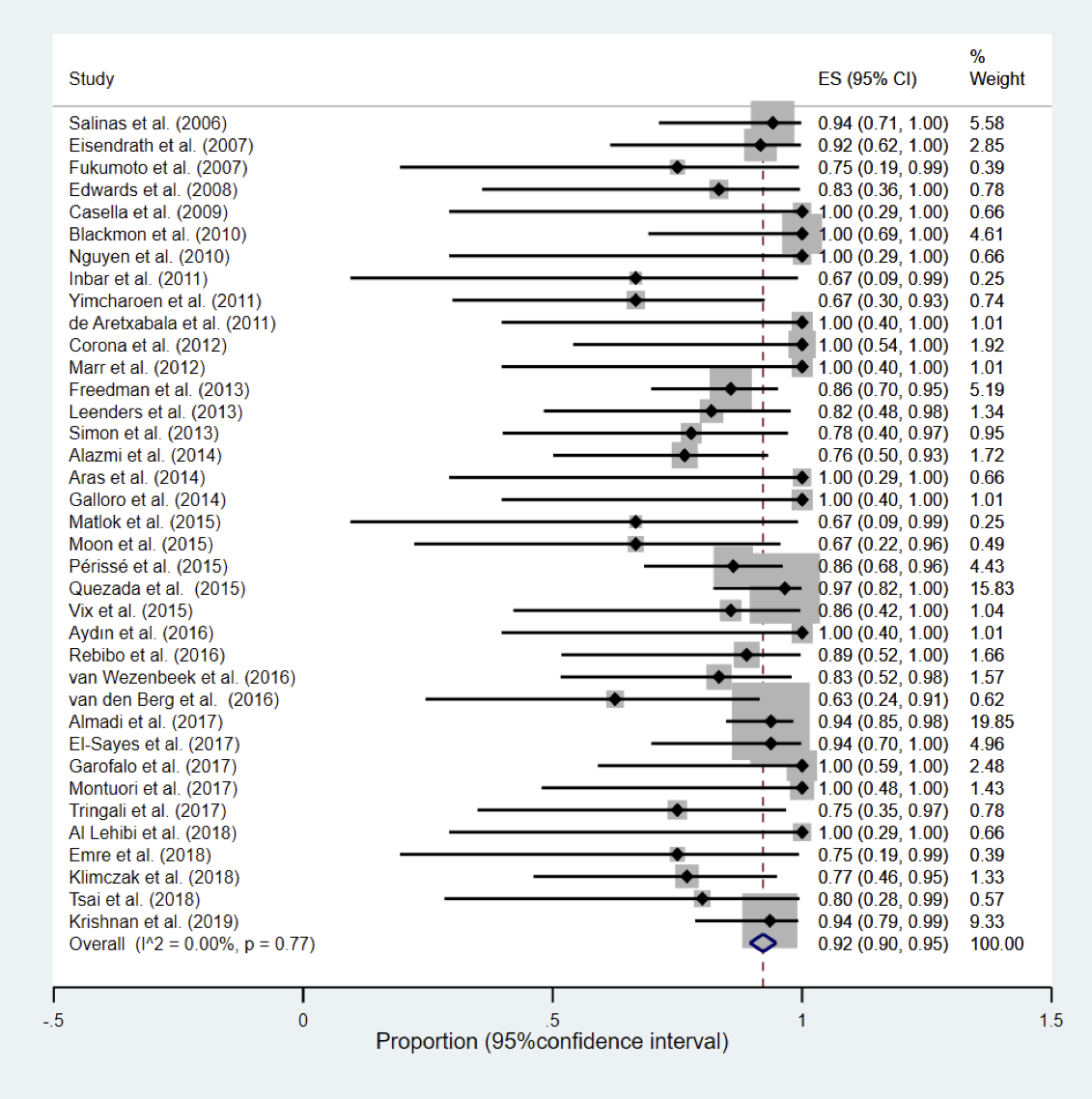

Supplement: Supplementary file 2 — Figure S2. Forest plot - successful leak closure after exclusion of outliers (PNG 468 kb) [file 464_2020_7471_MOESM2_ESM.png]

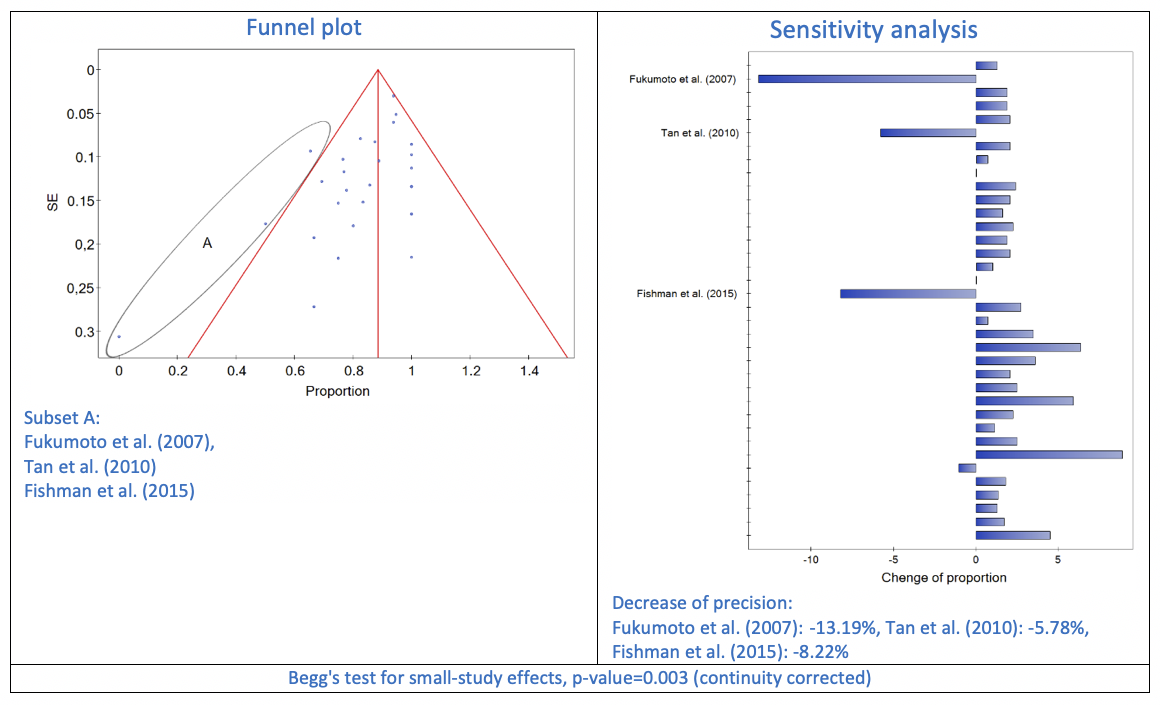

Supplement: Supplementary file 3 — Figure S3. Funnel plot, sensitivity analysis, and the Begg’s test result for successful leak closure in gastric sleeve group (PNG 248 kb) [file 464_2020_7471_MOESM3_ESM.png]

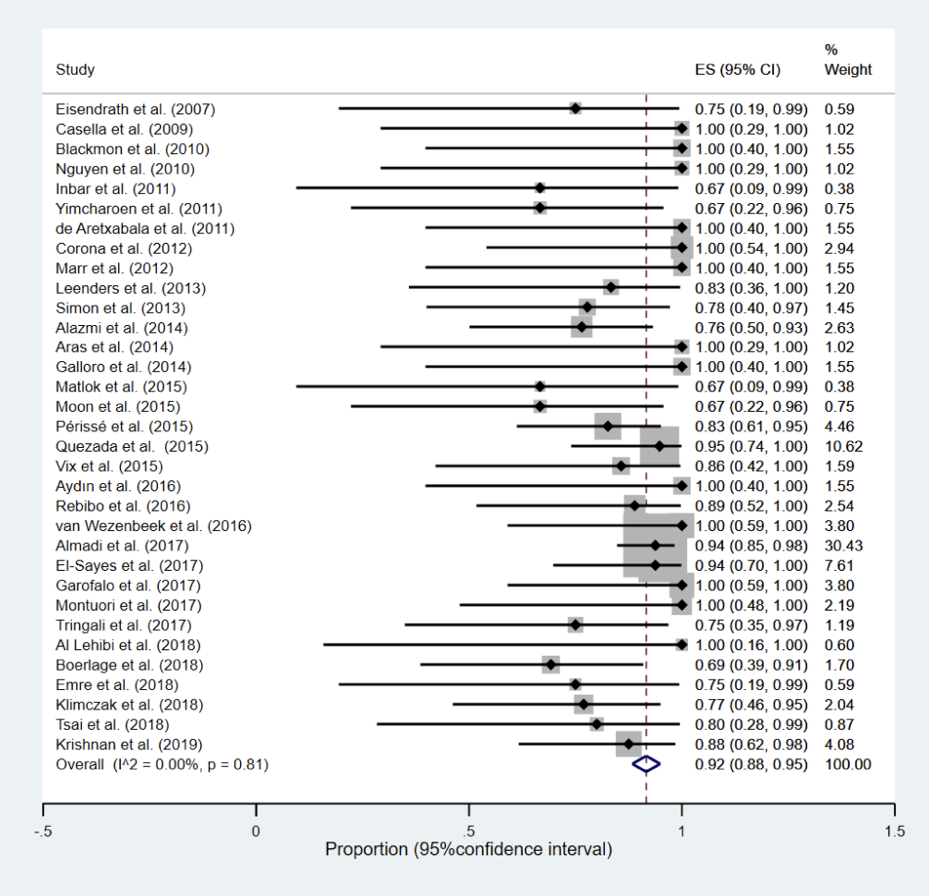

Supplement: Supplementary file 4 — Figure S4. Forest plot successful leak closure in gastric sleeve after exclusion of outliers (PNG 396 kb) [file 464_2020_7471_MOESM4_ESM.png]

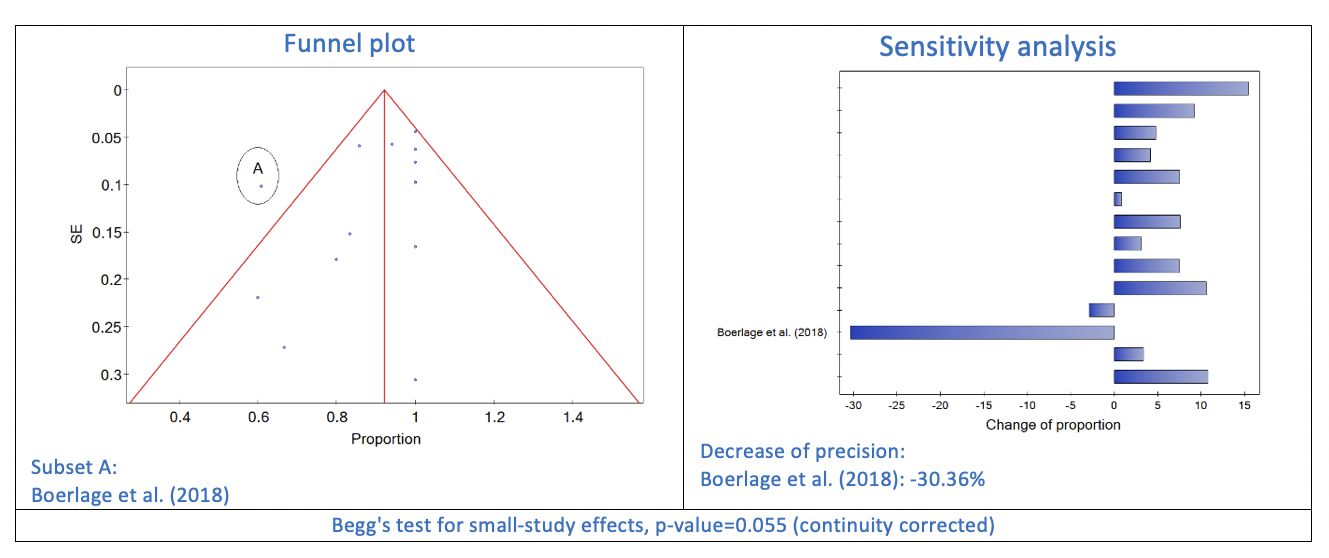

Supplement: Supplementary file 5 — Figure S5. Funnel plot, sensitivity analysis, and the Begg’s test result for successful leak closure in gastric bypass group (PNG 204 kb) [file 464_2020_7471_MOESM5_ESM.png]

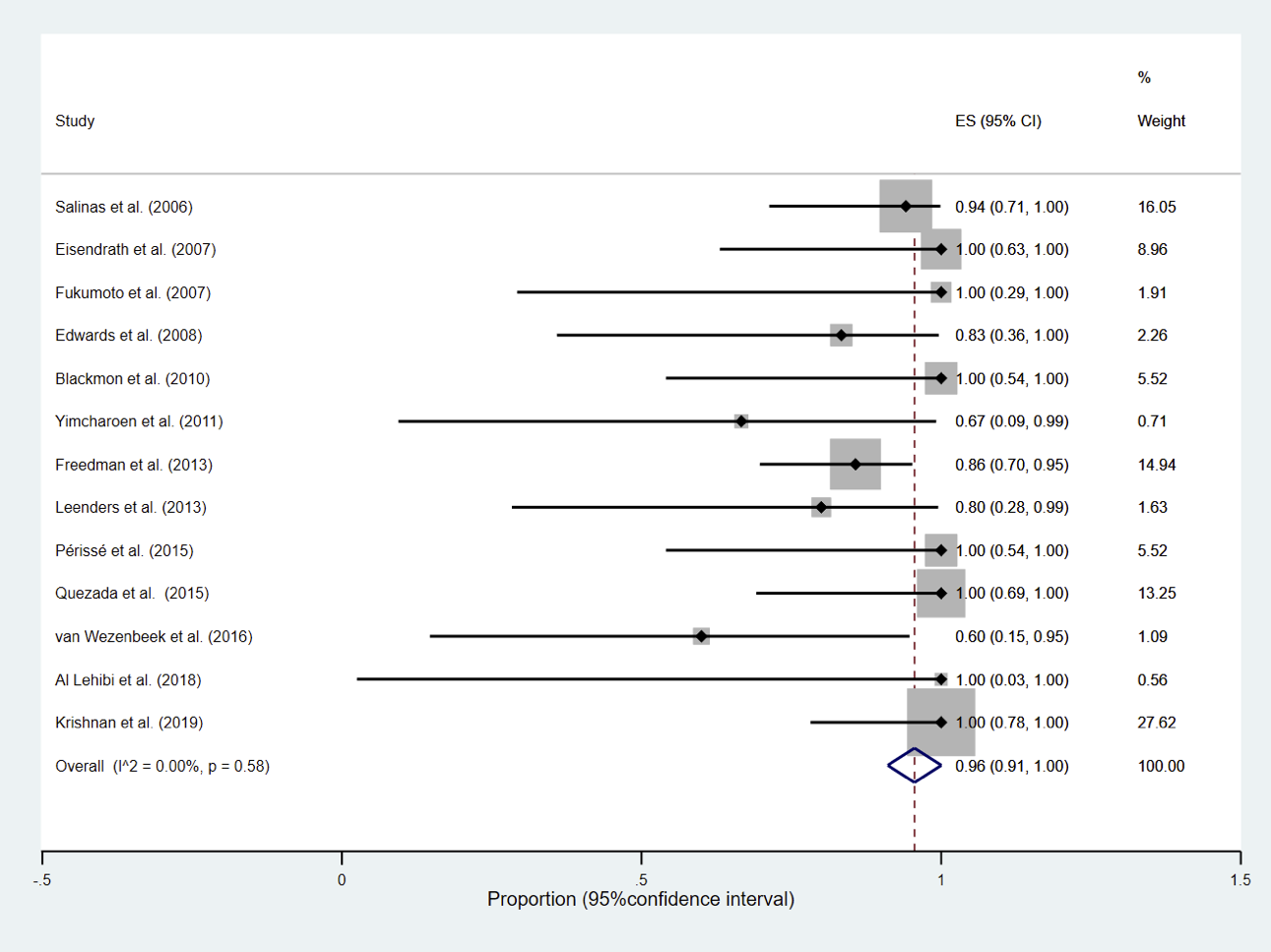

Supplement: Supplementary file 6 — Figure S6. Forest plot successful leak closure in gastric bypass group after exclusion of outliers (PNG 201 kb) [file 464_2020_7471_MOESM6_ESM.png]

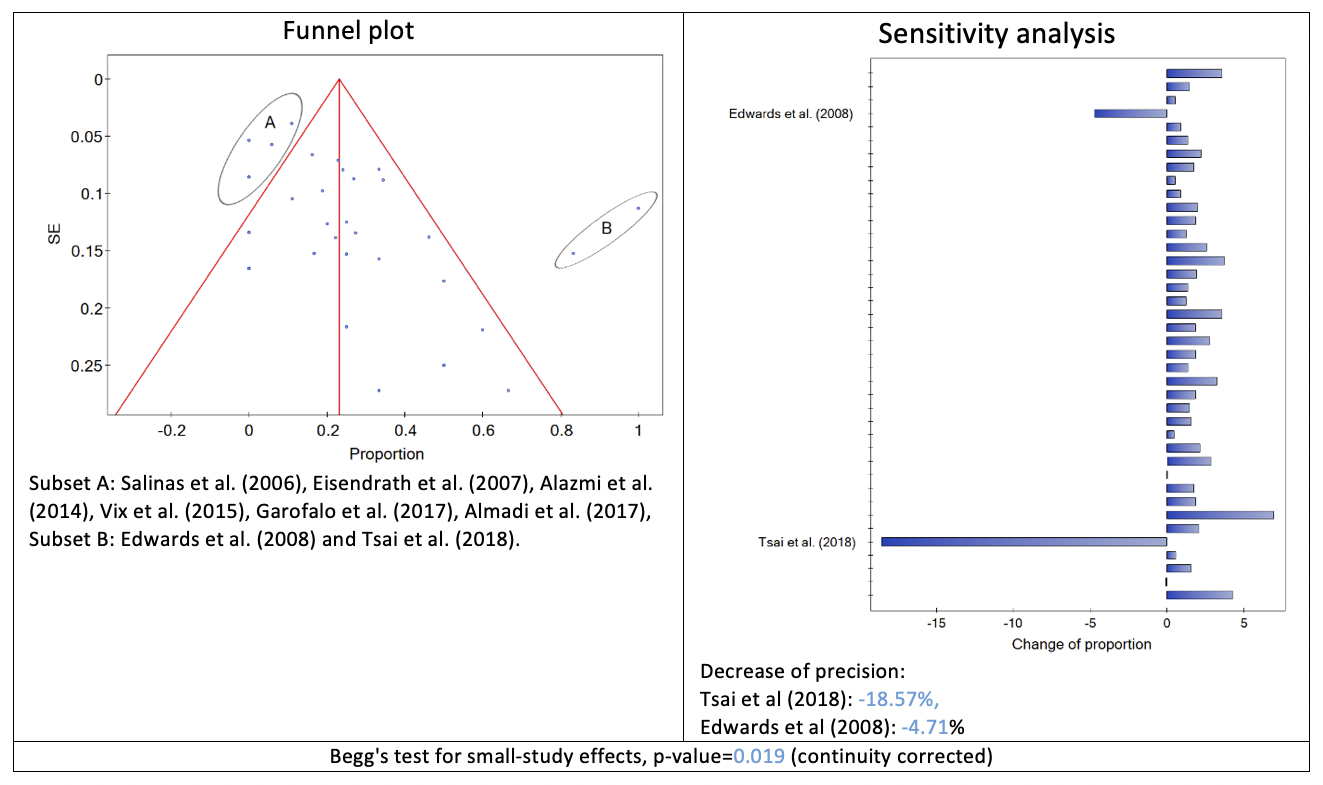

Supplement: Supplementary file 7 — Figure S7. Funnel plot, sensitivity analysis and the Begg’s test result for stent migration (PNG 280 kb) [file 464_2020_7471_MOESM7_ESM.png]

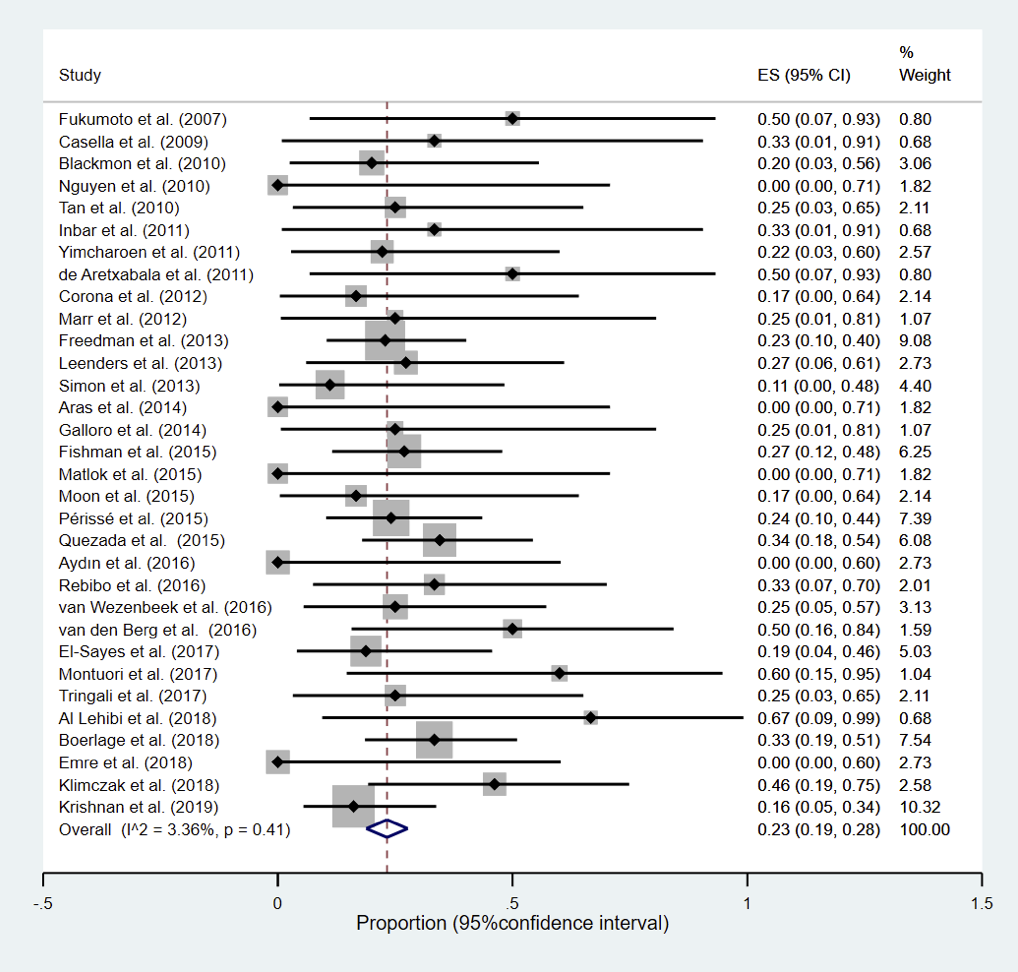

Supplement: Supplementary file 8 — Figure S8. Forest plot for stent migration after exclusion of outliers (PNG 383 kb) [file 464_2020_7471_MOESM8_ESM.png]

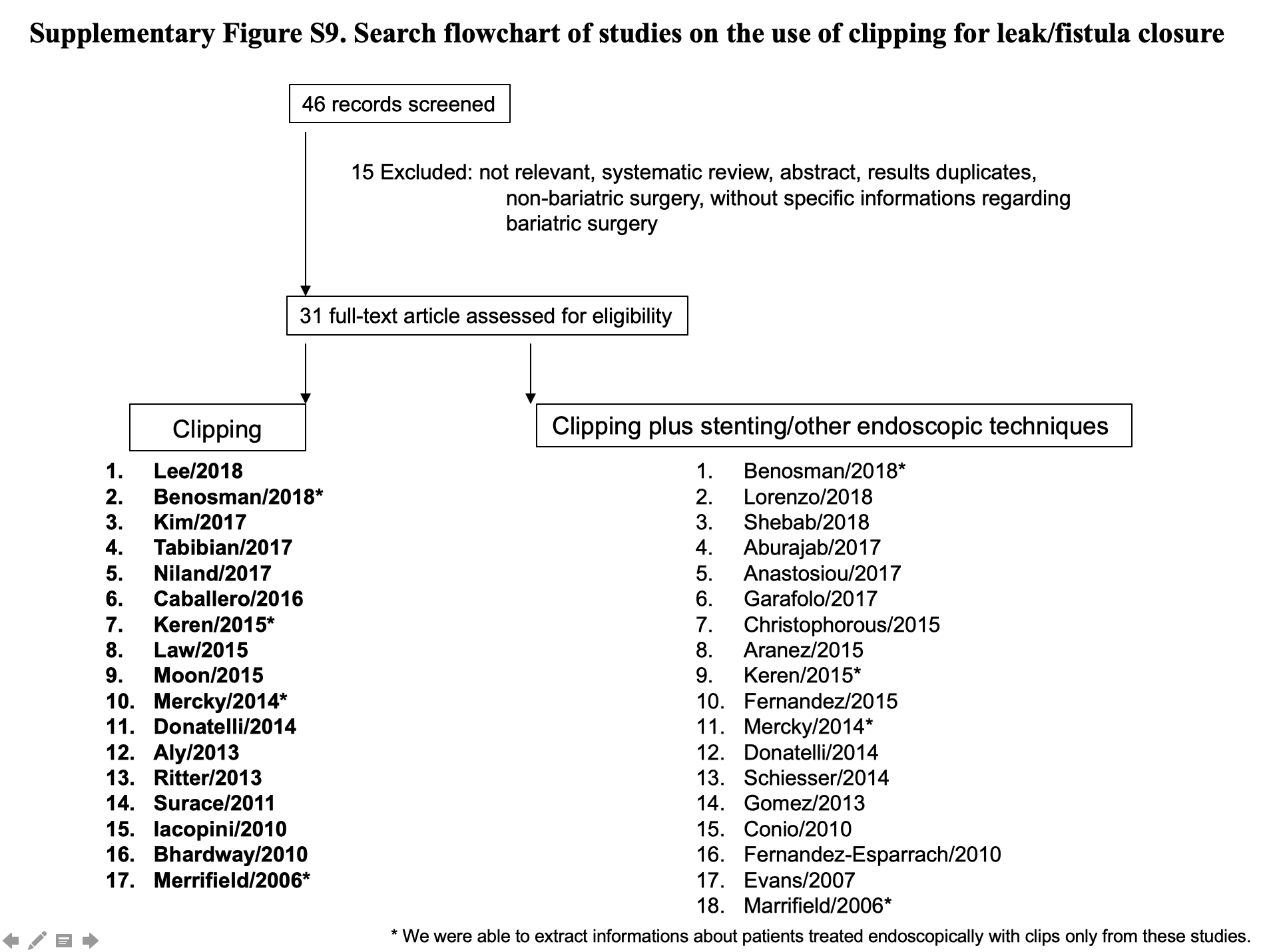

Supplement: Supplementary file 9 — Figure S9. Search flowchart of studies on the use of clipping for leak/fistula closure (PNG 309 kb) [file 464_2020_7471_MOESM9_ESM.png]
