# Supplementary material for: Endoscopic management of leaks and fistulas after bariatric surgery: a systematic review and meta-analysis
Source: Surg Endosc. 2020 Feb 27;35(3):1067–87. doi: 10.1007/s00464-020-07471-1 (PMC7886733; doi:10.1007/s00464-020-07471-1)
Supplement: Supplementary file 10 — Electronic supplementary material 2 (DOCX 63 kb) [file 464_2020_7471_MOESM10_ESM.docx]

**Supplementary materials.**

**Database search formula**

MEDLINE (PubMED)

((bariatric surgery) OR (bariatric surgeries) OR (bariatric operation) OR (bariatric operations) OR (bariatric procedure) OR (bariatric procedures) OR (bariatric surgical procedure) OR (bariatric surgical procedures) OR (obesity surgery) OR (metabolic surgery) OR (gastric bypass) OR (roux-en-y gastric bypass) OR (greenville gastric bypass) OR (gastroileal bypass) OR (gastrojejunostomy) OR (gastrojejunostomies) OR (gastroplasty) OR (gastroplasties) OR (collis gastroplasty) OR (vertical-banded gastroplasty) OR (vertical banded gastroplasty) OR (vertical-banded gastroplasty) OR (jejunoileal bypass) OR (jejuno-ileal bypasses) OR (ileojejunal bypass) OR (ileojejunal bypasses) OR (intestinal bypass) OR (intestinal bypasses) OR (biliopancreatic bypass) OR (biliopancreatic diversion) OR (duodenal switch) OR (pancreatobiliary bypass) OR (gastric banding) OR (stomach banding) OR (swedish gastric banding) OR (swedish adjustable gastric banding) OR (laparoscopic adjustable gastric banding) OR (laparoscopic adjustable silicone banding) OR (sleeve gastrectomy) OR (gastric sleeve)) AND ((stent) OR (clips) OR (clip) OR (clipping) OR (TTSC) OR (OTSC) OR (suturing) OR (suture) OR (stitch) OR (OverStitch) OR (tissue sealant) OR (biologic glue) OR (fibrin glue) OR (cyanoacrylate) OR (N-butyl-2-cyanoacrylate) OR (Histoacryl) OR (band ligation) OR (Amplatzer Septal Occluder) OR (cardiac septal defect occluder) OR (vacuum therapy) OR (Endo-SPONGE) OR (vacuum assisted) OR (vicryl mesh)) AND ((leak) OR (fistula) OR (complications)) AND ((endoscopy) OR (endoscopic) OR (endoscopically))

SCOPUS

({bariatric surgery} OR {bariatric surgeries} OR {bariatric operation} OR {bariatric operations} OR {bariatric procedure} OR {bariatric procedures} OR {bariatric surgical procedure} OR {bariatric surgical procedures} OR {obesity surgery} OR {metabolic surgery} OR {gastric bypass} OR {roux-en-y gastric bypass} OR {greenville gastric bypass} OR {gastroileal bypass} OR {gastrojejunostomy} OR {gastrojejunostomies} OR {gastroplasty} OR {gastroplasties} OR {collis gastroplasty} OR {vertical-banded gastroplasty} OR {vertical banded gastroplasty} OR {vertical-banded gastroplasty} OR {jejunoileal bypass} OR {jejuno-ileal bypasses} OR {ileojejunal bypass} OR {ileojejunal bypasses} OR {intestinal bypass} OR {intestinal bypasses} OR {biliopancreatic bypass} OR {biliopancreatic diversion} OR {duodenal switch} OR {pancreatobiliary bypass} OR {gastric banding} OR {stomach banding} OR {swedish gastric banding} OR {swedish adjustable gastric banding} OR {laparoscopic adjustable gastric banding} OR {laparoscopic adjustable silicone banding} OR {sleeve gastrectomy} OR {gastric sleeve}) AND ({stent} OR {clips} OR {clip} OR {clipping} OR {TTSC} OR {OTSC} OR {suturing} OR {suture} OR {stitch} OR {OverStitch} OR {tissue sealant} OR {biologic glue} OR {fibrin glue} OR {cyanoacrylate} OR {N-butyl-2-cyanoacrylate} OR {Histoacryl} OR {band ligation} OR {Amplatzer Septal Occluder} OR {cardiac septal defect occluder} OR {vacuum therapy} OR {Endo-SPONGE} OR {vacuum assisted} OR {vicryl mesh} OR {internal drainage} OR {septotomy}) AND ({leak} OR {fistula} OR {complications}) AND ({endoscopy} OR {endoscopic} OR {endoscopically}) AND ( LIMIT-TO ( PUBSTAGE,"final" ) ) AND ( LIMIT-TO ( DOCTYPE,"ar" ) ) AND ( LIMIT-TO ( SUBJAREA,"MEDI" ) ) AND ( LIMIT-TO ( LANGUAGE,"English" ) )
